# Insights into the Dynamic Succession of Microbial Community and Related Factors of Vanillin Content Change Based by High-Throughput Sequencing and Daqu Quality Drivers

**DOI:** 10.3390/foods12234312

**Published:** 2023-11-29

**Authors:** Wenhua Tong, Shuqin Wang, Ying Yang, Zhijiu Huang, Yiyun Li, Dan Huang, Huibo Luo, Liming Zhao

**Affiliations:** 1 School of Biological Engineering, Sichuan University of Science and Engineering, Yibin 644000, China; 2 Key Laboratory of Brewing Biotechnology and Application, Yibin 644000, China; 3 Sichuan Luzhou Laojiao Co., Ltd., Luzhou 646000, China; 4 Zuiqingfeng Distillery Co., Ltd., Luzhou 646000, China; 5 School of Biotechnology, East China University of Science and Technology, Shanghai 200000, China

**Keywords:** *Nongxiangxing Baijiu*, *Daqu*, vanillin, high-throughput sequencing, metabolize

## Abstract

*Daqu* is an important saccharifying starter in the fermentation of *Nongxiangxing Baijiu* in China. Vanillin is a health and flavor factor in Baijiu. However, only a few research studies on the vanillin content of *Daqu* are currently not systematic. In order to investigate the metabolic mechanism of vanillin in the fermentation process of *Daqu*, we analyzed the changes in microorganisms, influencing factors, and enzymes related to vanillin in *Daqu*. This research found that there were differences between bacterial and fungal genera in each sample, and the abundance of bacteria was greater than that of fungi. Among the microbial genera, *Klebsiella*, *Escherichia*, *Acinetobacter*, *Saccharopolyspora*, *Aerococcus*, and *Puccinia* were positively correlated with vanillin. Meanwhile, we also found that moisture and reducing sugar were the main physicochemical factors affecting the formation of vanillin. The functional annotation results indicate that carbohydrate metabolism and energy metabolism were important microbial metabolic pathways that impacted vanillin production in solid-state fermentation. The feruloyl-CoA hydratase/lyase (EC 4.1.2.61) and acylamidase (EC 3.5.1.4) were positively correlated with vanillin content (*p* ≤ 0.05) and promote the increase in vanillin content. These findings contribute to furthering our understanding of the functional microorganisms, physicochemical factors, and enzymes related to the change in vanillin content during the fermentation of *Daqu* and can help to further explore the flavor substances in *Baijiu* fermentation in the future.

## 1. Introduction

**Figure 1:**
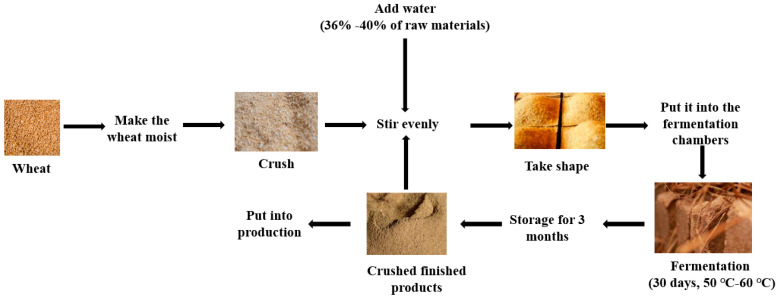
*Daqu* production process.

*Baijiu* is a strong liquor with a long history in China. According to different brewing techniques and flavor characteristics, it is divided into 12 flavors [1]. *Nongxiangxing Baijiu* refers to the *Baijiu* produced by fermentation, distillation, storage, aging, and blending with *Daqu* as the starter, grain as the raw material, and a mud cellar as the container [2]. It has the characteristics of a thick body, a mellow taste, and a rich aroma [3] and is widely welcomed by consumers. As a solid fermentation starter for *Baijiu*, *Daqu* is also an important raw material in the brewing process of *Nongxiangxing Baijiu* and largely determines the flavor and quality of the final *Baijiu* product [4]. The production of *Nongxiangxing Daqu* mainly uses wheat, barley, and other raw materials, grinding them, mixing them with water, manually stepping on them to form block-shaped *Daqu* embryos, and fermenting them at a certain temperature and humidity [5] (Figure 1). Depending on the starter-making temperature, *Daqu* can be divided into high-temperature *Daqu* (60–70 °C), medium–high-temperature *Daqu* (50–60 °C), medium-temperature *Daqu* (45–50 °C), and low-temperature *Daqu* (40–45 °C) [6]. *Nongxiangxing Baijiu Daqu* belongs to the medium–high-temperature *Daqu* group. Microorganisms are one of the factors affecting the flavors of *Baijiu*, and many studies have shown that microorganisms play an important role in *Baijiu* fermentation [7,8]. The quality of the *Daqu* is also related to microorganisms [9,10]. Microbial interactions lead to the formation of a stable brewing functional microbial community in the *Daqu* [11,12,13,14]. These microorganisms enable the raw materials to be converted into functional enzymes and flavor components during the fermentation process, to develop a certain flavor in the finished product [15]. Functional enzymes can further degrade large molecules, such as starch and protein, in raw materials into small molecules, promote the growth of microorganisms, provide energy for flavor metabolism, and make the final product have a good aroma [16]. At present, there are many studies using high-throughput sequencing technology combined with physicochemical characteristics to analyze the microbial community succession and environmental driving factors during the fermentation process of *Daqu* (Table 1).

Vanillin (4-hydroxy-3-methoxybenzaldehyde) is an important phenolic substance in Baijiu [23,24]. Tannin, xylogen, and protein in liquor-making raw materials are the main sources of vanillin in *Baijiu*. As a derivative of ferulic acid and an analog of guaiacol, vanillin has been detected in different types of *Baijiu* [25,26]. Vanillin also has various biological activities and can be used as a free radical scavenger [27]. It is widely used in different fields such as the food industry, the pharmaceutical industry, the tobacco industry, and so on [28,29]. There are three main preparation methods for vanillin [30]: the natural extraction method, the chemical synthesis method, and the microbial transformation method. Among them, the microbial transformation method is not affected by seasons and has almost the same aroma and purity as naturally extracted vanillin, which is widely used [31]. At present, the research on vanillin in *Baijiu* mainly focuses on the content of vanillin in each flavor type of the finished liquor, and there is no systematic study on the microorganisms related to vanillin production in *Daqu*.

This study determined the vanillin content during the fermentation process of *Nongxiangxing Baijiu Daqu* and identified the key points of vanillin content changes (0d, 8d, 13d, 21d, 27d, and 60d). We intended to investigate the changes in microorganisms, physicochemical factors, and enzymes related to vanillin metabolism and their correlation with the vanillin content during the fermentation process of *Daqu*. It is of great significance for furthering our understanding of the succession and metabolic pathways of vanillin microbial communities.

## 2. Materials and Methods

### 2.1. Sample Collection

*Daqu* samples were collected from a distillery in Yibin, China. Sampled at six different time points, Day 0, Day 8, Day 13, Day 21, Day 27, and Day 60, named D0, D8, D13, D21, D27, and D60, respectively. Among them, D60 was the sample stored for 30 days. Each batch of samples was taken from three different fermentation chambers, and one parallel sample consisted of five sub-samples from different locations in a single fermentation chamber to form three parallel samples. The samples were divided into two portions and stored in a sterile plastic bag; one part was stored at −80 °C for DNA extraction, and the other part was stored at −20 °C for physicochemical analysis.

### 2.2. Determination of Vanillin Content

A certain amount of vanillin standard was weighed and diluted with 50% ethanol to obtain the mother liquid. It was then gradually diluted to obtain a series of standard stock solutions. The sample was ground thoroughly, 5 mL of physiological saline was added, and it was shaken well and centrifuged (r/min, 10 min). The supernatant was taken and diluted appropriately with a 50% methanol solution after being passed through a 0.22 μm filter and stored for later use. The mobile phase was methanol: 0.05% glacial acetic acid (35:65). The mobile phase and ultrapure water were filtered and ultrasonically treated. Flow rate: 1 mL/min, detection wavelength: 260 nm, column temperature: 30 °C, injection volume: 10 μL, gradient elution. Analyses were based on retention time and peak area.

### 2.3. Analysis of Physicochemical Properties

The temperature of the sample and the temperature and humidity of the fermentation chambers were recorded. A certain amount of sample was taken, and the moisture of *Daqu* was determined by the direct drying method at 105 °C. An acidity meter was used to determine the pH of the sample. The acidity and amino acid nitrogen were determined by titration with 0.1 mol/L standard NaOH solution, and the final titration points of pH 8.2 and pH 9.2, respectively [22,32]. Proteins were removed from the samples, and the reducing sugar content was calculated from the volume of sample solution consumed during titration of the alkaline copper tartrate solution, using methylene blue as an indicator. The saccharifying power was determined according to general analytical methods (QB/T 4257-2011 Methods) [33].

### 2.4. DNA Extraction, Amplification, and Sequencing

Total DNA was extracted using CTAB method for samples [34]. And the DNA absorbance levels at wavelengths of 260 and 280 nm were measured with a NanoDrop2000 ultramicro spectrophotometer. The universal primers for bacterial 16S rDNA were 27f (AGAGTTTGATCCTGGCTCAG) and 1492r(TACCTTGTTACGACTT). The universal primers for fungal 18 S rDNA were ITS1(TCCGTAGGTGAACCTGCGG) and ITS4 (TCCTCCGCTTATTGATATGC). The PCR amplification program was performed by PCR equipment (TC-96/G/H(b)C, China). PCR amplification was performed as follows: 94 °C for 4 min, 94 °C for 30 s, 55 °C for 30 s, 72 °C for 45 s, 34 cycles, 72 °C for 5 min, ending at 25 °C. PCR products were separated on a 1.5% agarose gel, the E.Z.N.A.^®^ Gel & PCR Clean UP kit (OMEGA, Norcross, GA, USA) was used to purify, elute, and quantify. Library establishment and high-throughput sequencing were performed by Shanghai Majorbio Bio-Pharm Technology Co., Ltd. (Shanghai, China).

### 2.5. Processing of Raw Sequence Data

The raw data obtained from sequencing (Raw Data) have a certain proportion of low-quality data. In order to ensure the accuracy and reliability of the subsequent information analysis results, the software Fastp (v0.23.0, https://github.com/OpenGene/fastp (accessed on 8 September 2023)) was used to subtract the connector sequences at end 3‘ and end 5. Data were removed that had a length of less than 50 bp, an average base mass value of less than 20, and N bases after shearing, while data were retained that had high-quality pair-end reads and single-end reads. The splicing software MEGAHIT (v1.1.2, https://github.com/voutcn/megahit (accessed on 8 September 2023)) was used to gradually splice the segments with high- and low-sequencing depths after quality control, and contigs were selected with ≥300 bp from the splicing results as the final assembly result. Prodigall was used to pre dict ORF for contigs in the splicing results. Genes were selected with a nucleic acid length of ≥100 bp and translated into amino acid sequences. The predicted gene sequences of all samples were clustered using CD-HIT, and the longest gene was selected as the representative sequence for each class to construct a non-redundant gene set. SQAPaligner (soap2.21release, https://github.com/ShujiaHuang/SOAPaligner (accessed on 8 September 2023)) software was used to compare the high-quality reads of each sample with a non-redundant gene set (95% identity) and calculate the abundance information of genes in the corresponding samples. Diamond was used to compare the amino acid sequences of the non-redundant gene set with eggCOG, KEGG, and CAZy databases to obtain specific functional annotation information for the sample metagenome.

### 2.6. Data Analysis

All samples were measured three times, and the data show the standard deviation of the average of the three measurements. Analysis of similarities (ANOSIM) was utilized to determine whether the groupings were meaningful. The OTU abundance table was standardized using PICRUSt2. The richness and diversity of microbial community were calculated according to α-diversity. Statistical methods were used to conduct hypothesis testing on species between microbial communities and analyze differences in species and functions. Principal component analysis (PCA) was used to analyze the intergroup differences in samples from *Daqu*. Correlation analyses were investigated by heatmap diagrams and redundancy analysis (RDA) to show the relationship between the physicochemical properties and microorganisms at the genus level. Genes were annotated using Evolutionary genealogy of genes: Non-supervised Orthologous Groups (EggNOG, http://eggnog.embl.de/ (accessed on 8 September 2023)) [35], Kyoto Encyclopedia of Genes and Genomes (KEGG, http://www.genome.jp/kegg/ (accessed on 6 September 2023)) [36], and Carbohydrate-active enzymes (CAZy, http://www.cazy.org/ (accessed on 10 September 2023)) [37]. Based on KEGG annotation results, differential enzyme detection and visualization analysis of vanillin metabolic pathways were performed to obtain enzymes related to vanillin metabolism during fermentation. The raw sequence data reported in this paper have been deposited in the Genome Sequence Archive [38] in National Genomics Data Center [39], China National Center for Bioinformation/Beijing Institute of Genomics, Chinese Academy of Sciences (GSA: CRA012966), and are publicly accessible at https://ngdc.cncb.ac.cn/gsa (accessed on 12 October 2023).

## 3. Results

### 3.1. Vanillin Content and Physicochemical Properties during the Fermentation of Daqu

**Figure 2:**
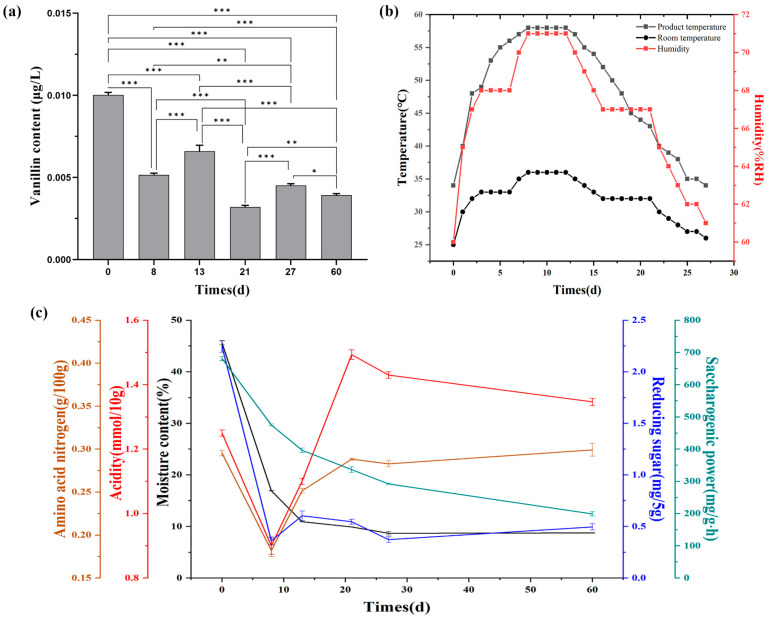
Changes in vanillin and physicochemical factors during *Daqu* fermentation process. Change in vanillin content (0.01 < *p* ≤ 0.05 *, 0.001 < *p* ≤ 0.01 **, *p* ≤ 0.001 ***) (**a**), changes in temperature and humidity during the fermentation process of *Daqu* (**b**), changes in physicochemical factors of *Daqu* (**c**).

The determination of vanillin content during *Daqu* fermentation revealed significant changes in vanillin content between days 0, 8, 13, 21, and 27 of *Daqu* fermentation and 30 days of storage (Figure 2a), and the differences were significant. Among them, the content of vanillin decreased significantly from D0 to D8 and from D13 to D21. From D8 to D13 and from D21 to D27, the vanillin content increased. To investigate the factors that affect the vanillin content during the fermentation process of *Daqu*, the temperature and humidity changes during the fermentation process were recorded. The physicochemical properties of samples at six time points were measured, and significant difference tests were conducted.

The temperature, humidity, and *Daqu* temperature of the curved room first slowly rose, then stabilized and ultimately decreased (Figure 2b), which is in line with the characteristics of “front slow, middle stiff, and back slow falling” in *Daqu* fermentation [40]. The physicochemical properties’ measurement results for each key point are shown in Figure 2c. The moisture and saccharification power showed a continuous downward trend throughout. The moisture content of *Daqu* decreased from 42.59 ± 0.35% to 8.66 ± 0.35%, and the saccharification power of *Daqu* decreased from 681 ± 6.08 mg/g·h to 199 ± 6.55 mg/g·h. This is related to the temperature of *Daqu* and the temperature of the starter room. High-temperature conditions can inhibit or kill microorganisms related to saccharifying enzymes’ metabolism, leading to a decrease in saccharification power [41]. From D0 to D13, the trends of reducing sugars, acidity, and amino acid nitrogen were the same as those of vanillin. At D8–D13, a rise in the reducing sugar content of the macrobiotic fermentation was associated with a decrease in saccharification power. The acidity in *Daqu* mainly comes from the metabolism of organic acids and microorganisms in the raw materials [7]. As the fermentation proceeds (D13–D21), the microbial metabolism is vigorous, and the organic acids, reducing sugars, and other substances in the raw materials are decomposed [42]; however, the acidity (1.10 ± 0.01 mmol/10 g to 1.49 ± 0.02 mmol/10 g) and ammonia nitrogen (0.25 ± 0.002 g/100 g to 0.28 ± 0.001 g/100 g) content of the *Daqu* continues to rise. From D21 to D27, the increase in the concentration of alcohol and acidity inhibits the activity of starch hydrolyzing enzymes, which results in a decrease in the concentration of reducing sugars. In the later stages of fermentation, reducing sugar, acidity, and amino acid nitrogen content decreased. Upon entering the storage period, the physicochemical factors tend to be moderate.

### 3.2. The Richness and Diversity of Microorganisms during the Fermentation Process of Daqu

**Figure 3:**
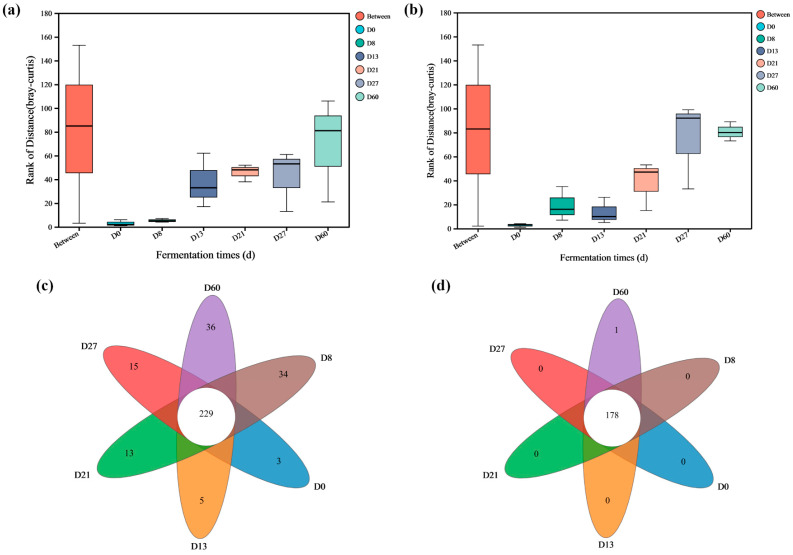
The richness and diversity of microorganisms during the fermentation process of *Daqu*. ANOSIM of bacteria (**a**), ANOSIM of fungi (**b**), Venn diagram of bacteria (**c**), Venn diagram of fungi (**d**). In (**a**,**b**), the “Between” boxes refer to differences between subgroups, while the others represent differences within the respective groups.

In order to explore the microorganisms related to vanillin, the microbial structure and species composition of each key point were analyzed. ANOSIM of microorganisms showed that inter-group differences were greater than intra-group differences (R values were 0.6367 and 0.5745, respectively), and the reliability of the test was high (*p* < 0.05), indicating that sample grouping was meaningful (Figure 3a,b). As shown in Figure 3c,d, at a 97% similarity level, 229 bacterial and 14 fungal OTUs were found in all six samples. And the fungal community is less complex than the bacterial community in the brewing process of *Nongxiangxing Baijiu Daqu*. Moreover, the unique OTU numbers of fungi decreased during the brewing process (Figure 3c,d).

The α-diversity can reflect the overall microbial abundance and diversity of *Daqu* samples. From D0 to D8, the Chao1 index and Shannon index of microorganisms increase, indicating an increase in microbial richness and diversity. As fermentation progresses, the abundance of microorganisms gradually decreases (Table 2). The diversity of bacteria and fungi reached their maximum values at D27 and D13, respectively. It is worth noting that, during the entire fermentation process of *Daqu*, the abundance of bacteria is greater than that of fungi. In addition, after one month of storage, there were significant changes in the microbial abundance and diversity of microorganisms, indicating that the relative concentration of core microorganisms tended to stabilize.

### 3.3. Influence of Microbial Diversity and Change on Vanillin Content

**Figure 4:**
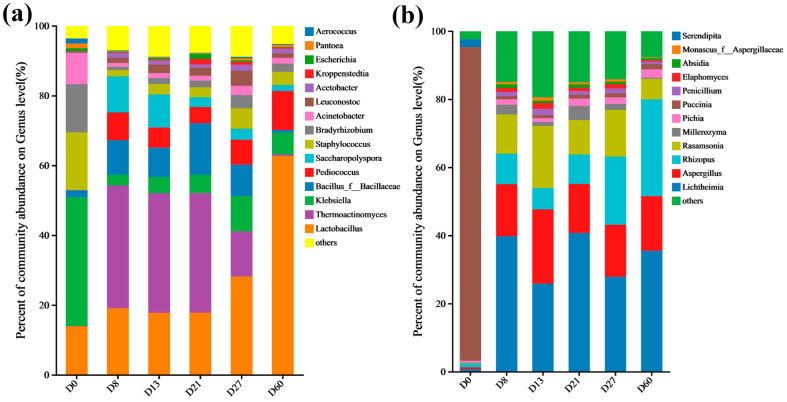
Community composition at the level of bacterial genus (**a**) and fungal genus (**b**) during the fermentation process of *Daqu* (the relative abundance ≥ 1%).

The main bacterial phyla included *Firmicutes*, *Protecobacteria*, and *Actinobacteria* (relative abundance > 1%) (Figure S1A). The main bacterial genera were *Lactobacillus*, *Thermoactinomyces*, *Klebsiella*, *Staphylococcus*, *Bacillus*, and *Pediococcus* (Figure 4a). Among them, *Lactobacillus* and *Bacillus* have been proven to be able to convert isoeugenol or ferulic acid to vanillin [43,44]. Throughout the fermentation phase, the relative abundance of *Lactobacillus* increased from 13.86% to 62.69%. The relative abundance of *Klebsiella* decreased from 36.96% to 2.95% from D0 to D8 and slightly increased from D8 to D27. After entering the storage period, it decreased from 10.12% to 6.25%. The abundance of *Bacillus* was only 1.93% at D0, slightly increased from D0 to D8, and then there was a downward trend from D8 to D13 and from D21 to D27. The relative abundance of *Thermoactinomyces* was almost 0% at D0, reached 34.27% at D8, and gradually stabilized at D21. The relative abundance of *Pediococcus* increased from 0.00% to 7.93% from D0 to D8, and there was a slight decrease from D8 to D13. An increasing trend from D21 to D27 reached 11.14% after one month of storage. This indicates that a certain amount of *Klebsiella* has a promoting effect on the generation of vanillin content, while *Bacillus* and *Thermoactinomyces* are not conducive to the generation of vanillin. In addition, the relative abundance change in *Staphylococcus* and *Acinetobacter* was consistent with the trend of vanillin content change, and they had an influence on the formation of vanillin.

The main fungal phyla included *Mucoromycota*, *Ascomycota*, and *Basidiomycota* (relative abundance > 1%) (Figure S1B). The main fungal genera were *Lichtheimia*, *Aspergillus*, *Rhizopus*, *Rasamsonia*, *Millerozyma*, and *Pichia* (Figure 4b). The relative abundance of *Aspergillus* increased from 0.55% to 21.79% from D0 to D13, and the relative abundance from D21 to D60 also slightly increased. The relative abundance of *Rhizopus* and *Rasamsonia* reached their maximum values during fermentation at D27 (19.92%) and D13 (18.82%), respectively. The relative abundance of *Rasamsonia* showed an upward trend from D21 to D27 (10.12% to 13.76%). A certain amount of *Aspergillus* and *Rasamsonia* had a promoting effect on the generation of vanillin. As early as 1996, the method of using *Aspergillus* to transform ferulic acid to produce vanillin appeared [45]. The abundance changes in *Lichtheimia*, *Millerozyma*, and *Pichia* were opposite to the changes in vanillin content, indicating that *Lichtheimia*, *Millerozyma*, and *Pichia* were not conducive to the generation of vanillin. After entering the storage period, the relative abundance of *Millerozyma* and *Rasamsonia* decreased, while the relative abundance of *Rhizopus*, *Lichtheimia*, and *Pichia* increased.

### 3.4. Analysis of Species and Functional Differences in Daqu Fermentation Process

**Figure 5:**
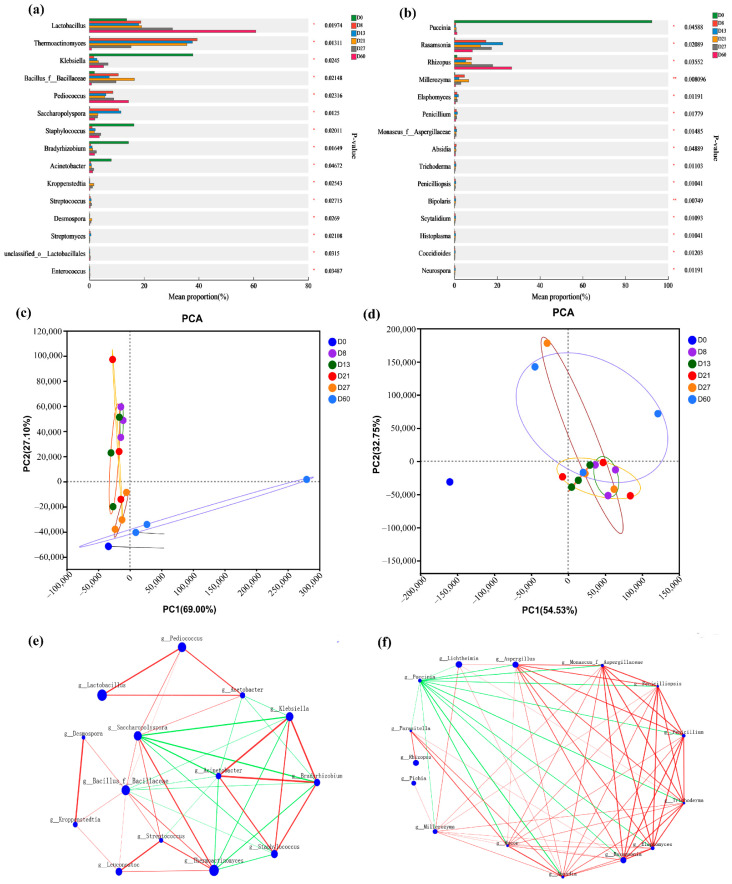
Analysis of species and functional differences in *Daqu* fermentation process. Intergroup difference test for bacteria (**a**) and fungi (**b**) (0.01 < *p* ≤ 0.05 *, 0.001 < *p* ≤ 0.01 **). Principal Component Analysis of Bacteria (**c**) and Fungi (**d**), analysis of species correlation network between bacteria (**e**) and fungi (**f**).

Analysis of species and functional differences at key points of samples is conducive to a clearer understanding of the similarities and differences in microorganisms in samples. The PC1 and PC2 of the bacterial community explained a variance of 73.36% (Figure 5c), and the D60 sample showed significant differences in bacteria compared to the previous fermentation stage samples. The two coordinate axes PC1 and PC2 of the fungal community explain a variance of 87.31% (Figure 5d), and there is a significant difference between the fungi in the D0 sample and the fungi in the other five stages of the sample. This may be due to the gradual stabilization of interactions between microorganisms during fermentation. There are significant differences in relative abundance levels among the six stages, but the composition of dominant genera fluctuates little.

We used the obtained abundance data and Kruskal–Wallis’ rank sum test to detect species differences in microbial communities across samples (top 15 in total abundance) (*p* < 0.05). There are differences between bacterial and fungal genera in each sample, and different microorganisms change over fermentation time (Figure 5a,b). In the genus Fungi, the difference between different samples of *Millerozyma* and *Bipolaris* was significant (0.001 < *p* ≤ 0.01). At the same time, the correlation and distribution of major microbial genera were analyzed through species distribution network analysis (Figure 5e). Among them, *Lactobacillus* is positively correlated with *Pediococcus* and *Acetobacter*. *Saccharopolyspora* is positively correlated with *Bacillus*, *Pediococcus*, *Acetobacter*, *Thermoactinomyces*. With the exception of Klebsiella and Acetobacter, all other microorganisms were positively correlated with Bacillus. *Puccinia* is negatively correlated with other fungi, while there is a positive correlation between other fungi. *Millerozyma* has a weak correlation with other fungi, while *Rasamsonia* has a strong correlation with other fungi. *Rhizopus* was only positively correlated with *Parasitella* and not with other fungi (Figure 5f).

### 3.5. Correlation Analysis between Microorganisms and Physicochemical Properties

**Figure 6:**
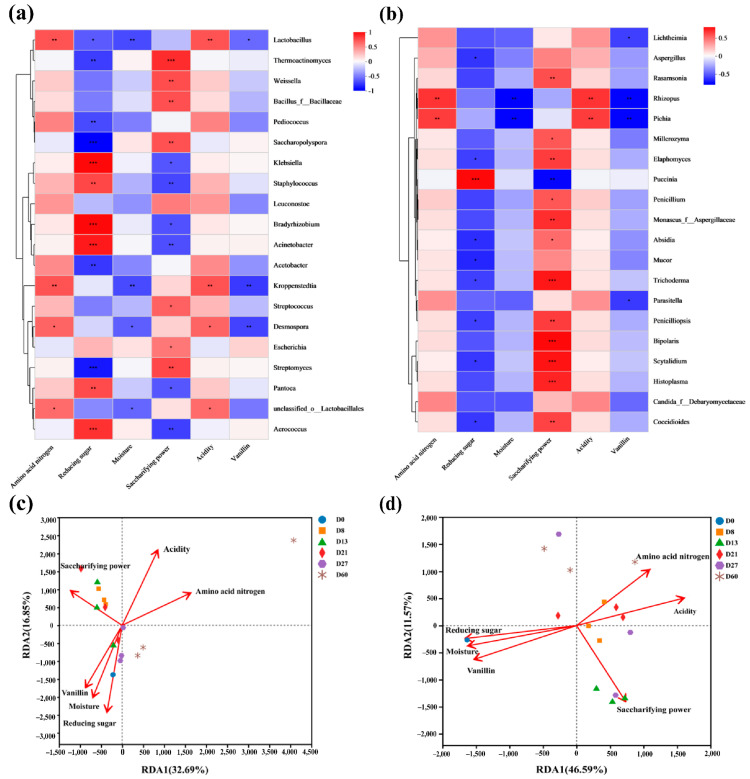
Correlation analysis between microorganisms and physicochemical properties (0.01 < *p* ≤ 0.05 *, 0.001 < *p* ≤ 0.01 **, *p* ≤ 0.001 ***). Heatmap of bacteria and physicochemical correlation (**a**), heatmap of fungi and physicochemical correlation (**b**), RDA analysis of bacteria (**c**), RDA analysis of fungi (**d**).

The changes in physicochemical factors and vanillin content are related to microorganisms. Bacterial genera microorganisms have varying degrees of promoting effects on amino acid nitrogen and acidity. *Lactobacillus* and *Kroppenstedtia* are positively correlated with amino acid nitrogen and acidity (0.001 < *p* ≤ 0.01) (Figure 6a). *Klebsiella*, *Bradyrhizobium*, *Acinetobacter*, and *Aerococcus* are significantly positively correlated with reducing sugar (*p* ≤ 0.001). On the contrary, *Saccharopolyspora* and *Streptomyces* are significantly negatively correlated with reducing sugar (*p* ≤ 0.001), so they are not conducive to the generation of reducing sugar. There is a negative correlation between *Lactobacillus* and *Kroppenstedtia* and moisture content. *Klebsiella*, *Escherichia*, *Acinetobacter*, *Saccharopolyspora*, and *Aerococcus* are positively correlated with vanillin (Figure 6a). At the level of fungal genera, microorganisms also have varying degrees of promoting effects on amino acid nitrogen and acidity. *Rhizopus* and *Pichia* show a positive correlation with amino acid nitrogen and acidity (0.001 < *p* ≤ 0.01) (Figure 6b). *Puccinia* and *Rhizopus* are positively correlated with reducing sugar and negatively correlated with saccharifying power, while other fungi are negatively correlated with reducing sugar (Figure 6b). *Trichoderma*, *Bipolaris*, *Scytalidium*, and *Histoplasma* show a significant positive correlation with saccharification power (*p* ≤ 0.001). Furthermore, *Puccinia* is significantly positively correlated with vanillin.

RDA results showed that two axes (RDA1 and RDA2) explained 32.69% and 16.85% of the total variance in bacteria (Figure 6c), indicating that these two axes can explain about 50% of the total variation in the sample bacterial community. The two axes of the sample fungi explain 46.59% and 11.57% of the total variance of the fungi, respectively (Figure 6d); these two axes can explain 58.16% of the sample fungal community variation. The relationship between sample RDA1 and RDA2 is shown in Table 3. RDA analysis of the correlation coefficient and significance test value of various environmental factors on the ranking results. For the bacterial community, reducing sugar and saccharifying power are significantly related to the two axes of RDA (*p* < 0.05), among which saccharification capacity (R^2^ = 0.4191) is the main factor affecting bacterial communities. For the sample fungal community, all physicochemical properties are significantly correlated with the two axes of RDA (*p* < 0.05). Acidity is the main factor affecting the fungal community of the sample (R^2^ = 0.8402), followed by reducing sugar (R^2^ = 0.8265) and moisture (R^2^ = 0.8138). In addition, vanillin, moisture, and reducing sugar are positively correlated with each other, while they are negatively correlated with acidity, saccharification power, and amino acid nitrogen.

### 3.6. Annotations on Species and Functions during the Fermentation Process of Daqu

**Figure 7:**
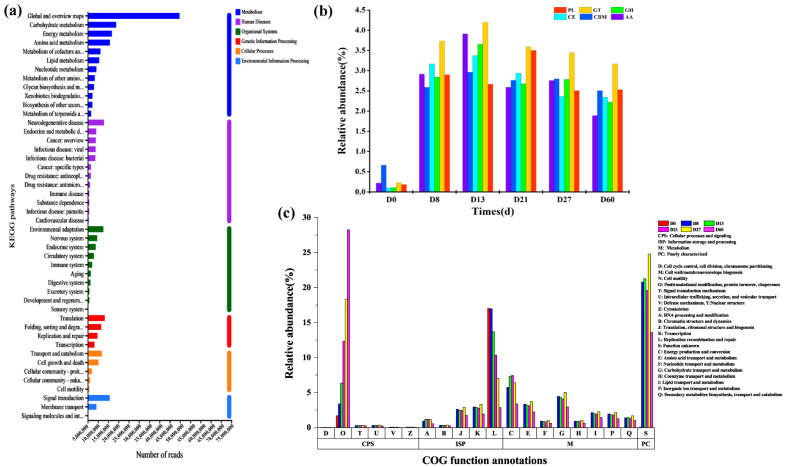
Annotations on species and functions during the fermentation process of *Daqu*. KEGG Function Annotations (**a**), CAZy Carbohydrate Activity Enzyme Annotation (**b**), EggCOG Function Annotations (**c**).

In order to explore the functional characteristics and differences in microbial populations at key points of production of *Daqu* and vanillin, the KEGG, CAZy, and eggNOG databases were used to annotate the metagenomic data. According to the KEGG database’s Level 1 annotation, a total of six types of metabolic pathways were annotated, with the highest number of genes annotated to metabolic processes and the second highest to human diseases. The genes annotated to environmental information factors are the least. As shown in Figure 7a, there are 46 types of Level 2 metabolic pathways in the KEGG database, with global and overview maps (20.35%) being the most abundant, followed by carbohydrate metabolism (6.3%), energy metabolism (5.35%), amino acid metabolism (4.91%), signal translation (4.87%), and translation (3.76%). Analysis based on KEGG indicates that the microbial community of *Daqu* is rich in genes involved in carbohydrate metabolism and energy metabolism. There may be many unknown functional genes in *Daqu* microorganisms that require further exploration and research.

According to the CAZy database, there are six carbohydrate-related enzymes annotated in the *Daqu* microbial genome, including glycoside hydrolase (GH), glycosyltransferase (GT), carbohydrate-binding module (CBM), glucoesterase (CE), auxiliary activities (AA), and polysaccharide lyase (PL). During the fermentation process of *Daqu*, the relative abundance of GT was the highest (18.37%), followed by GH (14.29%) and CBM (14.24%) (Figure 7b). The fermentation of *Daqu* is inseparable from the synthesis and decomposition of sugars. GT is a key enzyme in the synthesis of glycoside compounds from natural products [45,46]. The relatively high abundance indicates that there may be microorganisms in *Daqu* that can produce polysaccharides. The high relative abundance of GH may be due to the important role of GH in the hydrolysis and synthesis of biological sugars and glycoconjugates [47]. CBM plays an important role in the degradation of insoluble cellulose by cellulase [48]. *Daqu* is mostly made from grains, which contain various types of cellulose. The relatively high abundance of CBM can promote the degradation of cellulose in *Daqu* raw materials. CE includes ferulase, pectinesterase, etc., which can hydrolyze carbohydrate esters [49]. AA can assist other oxidoreductases in their functions, or assist GT in degrading lignocellulose [50]. During the fermentation process of *Daqu*, sugars, cellulose, and other substances in the raw materials are decomposed, and the relative abundance of various enzymes is also changed. Except for the relative abundance of PL reaching its maximum at D21, the relative abundance of other enzymes reaches its maximum at D13.

The corresponding COG annotations of the genes were obtained based on the EggNOG database (Figure 7c). The first three categories during the fermentation process of *Daqu* are O (posttranslational modification, protein turnover, chambers), S (function unknown), and L (replication, recombination, and repair). They belong to cellular processes and signal transduction, poorly characterized, information storage and process, respectively. The relative abundance of O-type genes gradually increases with fermentation, while the relative abundance of S-type genes is the highest in the later stage of fermentation (D27), reaching 13.59%, indicating that there are still many unknown genes that need to be further explored during the fermentation process of *Daqu*. The relative abundance of L-type genes gradually decreases with fermentation, indicating that the raw materials are gradually being utilized. C (energy production and conversion), E (amino acid transport and metabolism), and G (carbohydrate transport and metabolism) are closely related to cell metabolism [51], with minimal changes during the fermentation process, which is beneficial for the fermentation of *Daqu*.

### 3.7. Analysis of Enzymes and Related Factors Involved in Vanillin Metabolism

**Figure 8:**
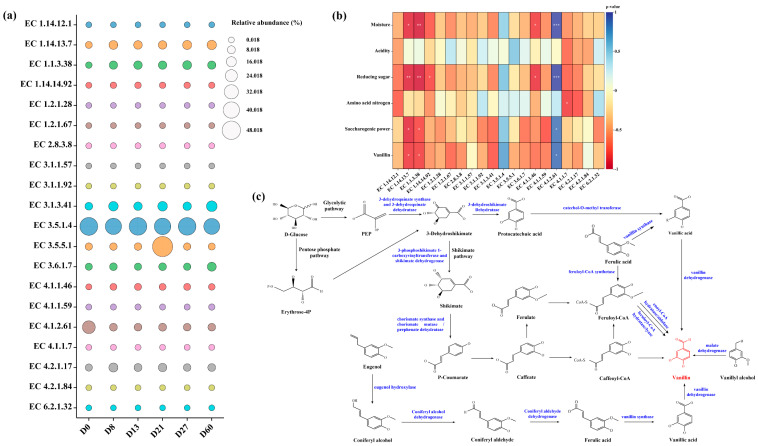
Changes in enzyme abundance during different fermentation stages (**a**), heatmap of correlation between enzymes and physicochemical factors during fermentation process (0.01 < *p* ≤ 0.05 *, 0.001 < *p* ≤ 0.01 **, *p* ≤ 0.001 ***) (**b**), synthesis pathway of vanillin (**c**), (Blue: name of enzyme; black: name of compound and related reaction; red: target compound).

As shown in Figure 8c, vanillin synthesis mainly has the following pathway: ferulic acid synthesizes vanillin through the action of feruloyl-CoA synthase [EC 6.2.1.34] and enoly-CoA hydratase/aldolase [EC 4.2.1.17]. Eugenol is oxidized by eugenol hydroxylase (ehyA and ehyB genes), coniferyl alcohol dehydrogenase [EC 1.1.1.90] and coniferyl aldehyde dehydrogenase [EC 1.2.1.68] to generate ferulic acid, which is then converted to vanillin [27,52,53,54]. D-glucose is processed to produce vanillin via the glycolytic pathway, pentose phosphate pathway, and shikimate pathway by a variety of enzymes, including 3-dehydroquinate synthase [EC 4.2.3.4], 3-phosphoshikimate-1-carboxyvinyltransferase [EC 2.5.1.19], feruloyl-CoA hydratase/lyase [EC 4.1.2.61]. Vanillin is produced via the glycolysis, pentose phosphate, and manganic acid pathways in the presence of various enzymes [55].

Through differential enzyme detection and visualization analysis of the vanillin metabolic pathway, the enzyme abundance during different fermentation stages is represented by bubble plots (Figure 8a). As shown in Figure 8a, there are certain differences in the relative abundance of various enzymes. In each sample, the relative abundance of acylamidase (EC 3.5.1.4) is high, and the trend of changes in this enzyme and vanillin content is consistent, with a positive correlation with changes in vanillin content. Furthermore, the relative abundance of feruloyl-CoA hydratase/lyase (EC 4.1.2.61) significantly decreased from D0 to D8, which is related to a decrease in vanillin content. Acetonitrilase (EC 3.5.5.1) increased by 48.82% from D13 to D21, which is not conducive to the increase in vanillin content. Among these enzymes, feruloyl-CoA lyase (EC 4.1.2.61) and acylamidase (EC 3.5.1.4) are positively correlated with vanillin content (*p* ≤ 0.05) (Figure 8b). The trends of 4-nitrophenylphosphotase (EC 3.1.3.41), acylphosphatase (EC 3.6.1.7), and enoyl-CoA hydratase (EC 4.2.1.17) were opposite to those of vanillin, which is not conducive to the generation of vanillin (Figure 8b).

## 4. Conclusions

This study found that there were differences among bacterial and fungal genera in each sample, and the abundance of bacteria was greater than that of fungi. *Lactobacillus*, *Thermoactinomyces*, *Klebsiella*, *Staphylococcus*, *Bacillus*, and *Pediococcus* were the main bacterial genera during the fermentation process of *Daqu*. *Lichtheimia*, *Aspergillus*, *Rhizopus*, *Rasamsonia*, *Millerozyma*, and *Pichia* were the main fungal genera. At the same time, among the microbial genera, *Klebsiella*, *Escherichia*, *Acinetobacter*, *Saccharopolyspora*, *Aerococcus*, and *Puccinia* were positively correlated with vanillin. Microbial changes were important factors affecting the formation of flavor substances. Through analyzing the relative abundance changes in microorganisms during fermentation, we found that *Bacillus*, *Thermoactinomycetes*, *Lichtheimia*, *Millerozyma*, and *Phchia* were not conducive to the production of vanillin. A certain amount of *Aspergillus* and *Rasamsonia* has a promoting effect on the generation of vanillin. *Klebsiella*, *Staphylococcus*, and *Acinetobacter* were positively correlated with the changes in vanillin content. In addition, we also found that moisture, saccharification power, and reducing sugar were the main physicochemical factors affecting the formation of vanillin. The carbohydrate metabolism and energy metabolism were the important microbial metabolic pathways that impacted vanillin production in solid-state fermentation. C (energy production and conversion), E (amino acid transport and metabolism), and G (carbohydrate transport and metabolism) were beneficial for the fermentation of *Daqu*. The feruloyl-CoA hydratase/lyase (EC 4.1.2.61) and acylamidase (EC 3.5.1.4) were positively correlated with vanillin content (*p* ≤ 0.05).

*Daqu* is a product of solid-state fermentation, which has the characteristics of open fermentation and multi-strain co-fermentation, and its flavor is affected by a variety of factors. We focused on exploring the metabolic mechanisms of health factors and flavor substances in the solid-fermentation system. In the future, research on functional microorganisms that produce vanillin can be carried out to explore specific metabolic mechanisms and functional genes, laying a solid foundation for the regulation and synthesis of vanillin in solid-state fermentation systems. Meanwhile, in actual production, the production process can be further regulated according to the changing characteristics of physicochemical factors, microbes, and enzymes, which ultimately achieve the purpose of improving the content of health factors in the solid-state fermentation system of *Daqu*.

## Figures and Tables

**Table 1:** Related articles on the analysis of microbial communities using high-throughput sequencing technology in the past four years.

Year of Publication	Related Colonies	Research Results
2020 [17]	*Firmicutes*, *Proteobacteria*, *Actinobacteria*, *Kroppenstedtia*, *Lactobacillus*, *Weissella*, *Lentibacillus*, *Bacillus*, *Saccharopolyspora*.	Firmicutes have significant advantages in the fermentation stage, *Kroppenstedtia*, *Lactobacillus*, *Weissella*, *Lentibacillus*, *Bacillus*, and *Saccharopolyspora* were detected as the main bacterial groups in the high-temperature *Daqu* of Chinese sesame-flavor liquor.
2021 [9]	*Lactic acid bacteria*, *Pichia pastoris*.	The results showed that microorganisms were obviously enriched, and the diversity of bacteria and fungi showed a downward trend during the heap fermentation process of Maotai-flavor Baijiu. However, the diversity of fungi in the pit fermentation process increased.
2021 [18]	*Thermoactinomyces*, *Lactobacillus*, *Saccharopolyspora*, *Bacillus*, *Streptomyces*, *Saccharomycopsis*, *Thermoascus*.	The different types of low-temperature *Daqu* had distinct flavor profiles, and the differences in the taste profiles were more significant. Dominated by *Thermoactinomyces* and *Lactobacillus*, together with *Saccharopolyspora*, *Bacillus*, *Streptomyces*, *Saccharomycopsis*, and *Thermoascus*, they formed the core microbiota that influenced the flavor of low-temperature *Daqu*. The bacteria mainly influenced the taste of low-temperature *Daqu*, whereas the fungi mainly influenced the aroma.
2022 [19]	*Saccharomycopsis*, *fibuligera*, *Debaryomyces hansenii*, *Lichtheimia ramosa*, *Lichtheimia corymbifera*, *Pichia kudriavzevii*.	The composition of the fungal community was similar, with *Saccharomycopsis fibuligera*, *Debaryomyces* hansenii, *Lichtheimia ramosa*, *Lichtheimia corymbifera*, and *Pichia kudriavzevii* being the most abundant and detected in most samples.
2022 [20]	*Lactococcus*, *Enterobacteriaceae*, *Lactobacillus*, *Weiss*.	The results showed that 39 different microbial genera were detected from *Daqu* samples of three storage periods, among which the dominant genera were *Lactococcus*, *Enterobacteriaceae*, *Lactobacillus*, and *Weiss*. The dominant microbial communities of *Daqu* vary greatly with different storage periods.
2023 [21]	*Lactobacillus*, *Stenotrophomonas*, *Firmicutes*, *Gammaproteobacteria*, *Actinobacteria*, *Streptomyces*, *Bordetella*, *Olivibacter*	During the initial fermentation stage, the bacterial community exerted a more pronounced effect on *Baijiu* quality than the fungal community. *Lactobacillus* was the dominant genus and biomarker in high-yield pit mud, and it constituted the only genus within the bacterial association network during the late fermentation stage.
2023 [22]	*Weissella*, *Pediococcus*, *Leuconostoc*, *Rhizopus*, *Staphylococcus*, *Alternaria*, *Weissella*, *Pediococcus*, *Burkholderia*.	In different sample groups of surface and central parts, the differential fungal genus was *Alternaria*, whereas differential bacteria genera were *Weissella*, *Pediococcus*, and *Leuconostoc*.

**Table 2:** Chao1 index and Shannon index in samples from *Daqu* fermentation process.

Samples	Bacteria		Fungi	
	Chao1	Shannon	Chao1	Shannon
D0	190.67	1.9763	124.00	0.4793
D8	789.67	2.1744	461.33	2.5040
D13	770.00	2.2579	459.00	2.7354
D21	754.67	2.2459	459.33	2.5659
D27	696.00	2.4055	453.00	2.4620
D60	666.00	1.7792	453.67	1.9592

**Table 3:** RDA analysis of the correlation coefficient and significance test value of various environmental factors on the ranking results.

	Bacteria				Fungi			
	RDA1	RDA2	*R* ^2^	*p*	RDA1	RDA2	*R* ^2^	*p*
Amino acid nitrogen	0.9661	0.2580	0.1846	0.2290	0.7781	0.6281	0.6455	0.0010
Reducing sugar	0.1665	−0.9860	0.3759	0.0450	−0.9773	−0.2117	0.8265	0.0010
Moisture	−0.0549	−0.9985	0.2516	0.1920	−0.9618	−0.2738	0.8138	0.0010
Saccharifying power	−0.7716	0.6362	0.4191	0.0160	0.4824	−0.8759	0.5482	0.0030
Acidity	0.1261	0.9920	0.2763	0.1490	0.9434	0.3316	0.8402	0.0010
Vanillin	−0.2448	−0.9696	0.1961	0.2270	−0.9217	−0.3878	0.8119	0.0020

## Data Availability

The data presented in this study are available on request from the corresponding author.

## Supplementary Materials

The following supporting information can be downloaded at: https://www.mdpi.com/article/10.3390/foods12234312/s1, Figure S1: Community composition at the level of bacterial phyla (**A**) and fungal phyla (**B**) during the fermentation process of *Daqu* (the relative abundance ≥ 1%).
