# Supplementary figures and images for: Insights into the Dynamic Succession of Microbial Community and Related Factors of Vanillin Content Change Based by High-Throughput Sequencing and Daqu Quality Drivers
Source: Foods. 2023 Nov 29;12(23):4312. doi: 10.3390/foods12234312 (PMC10705915; doi:10.3390/foods12234312)

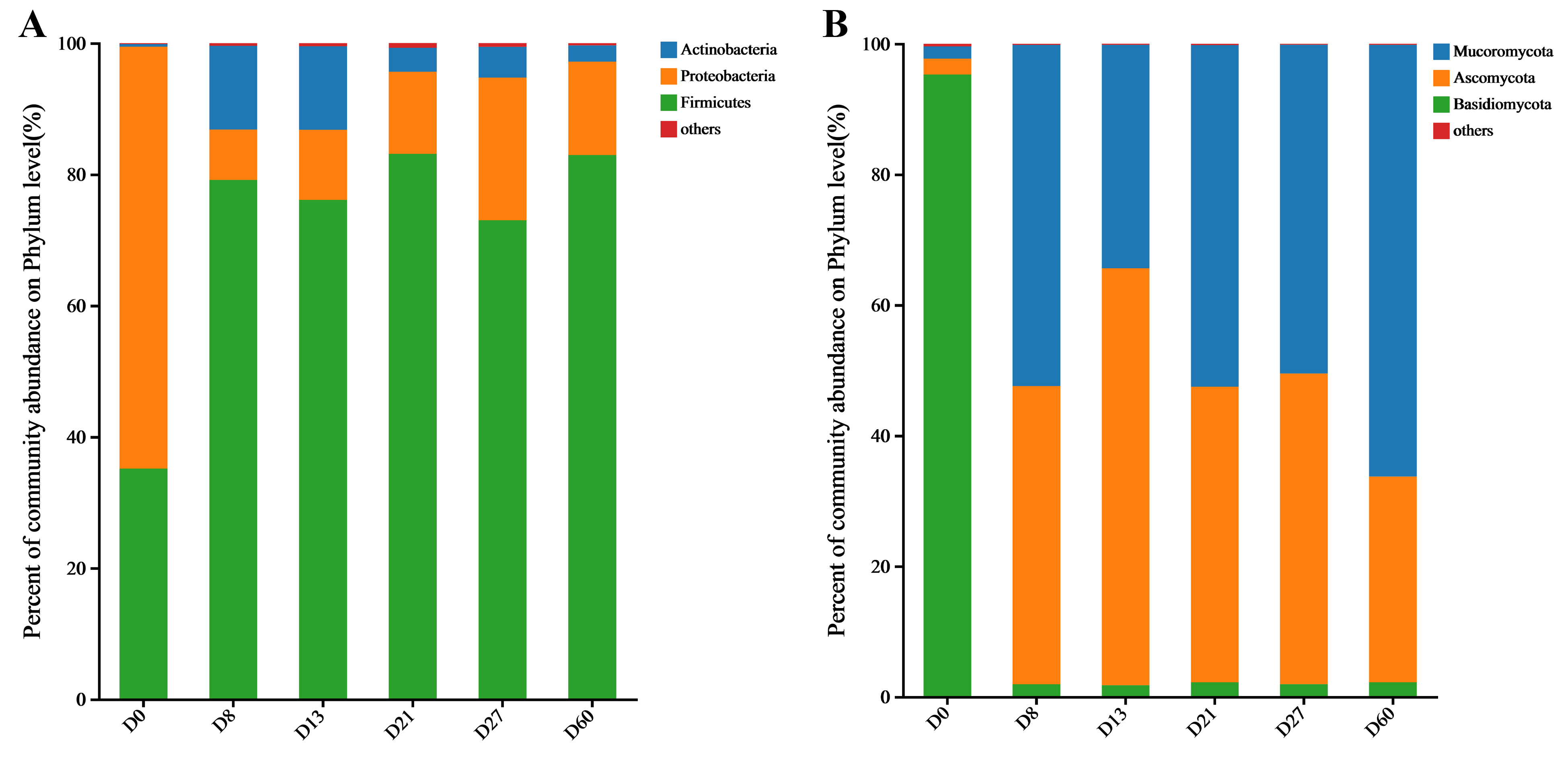

Supplement: Supplementary file 1 [file foods-12-04312-s001.zip › Fig. S1A.tif]
